# The Longitudinal Course of Gross Motor Activity in Schizophrenia – Within and between Episodes

**DOI:** 10.3389/fpsyt.2015.00010

**Published:** 2015-02-05

**Authors:** Sebastian Walther, Katharina Stegmayer, Helge Horn, Luca Rampa, Nadja Razavi, Thomas J. Müller, Werner Strik

**Affiliations:** ^1^University Hospital of Psychiatry, University of Bern, Bern, Switzerland

**Keywords:** actigraphy, psychosis, negative symptoms, PANSS, avolition

## Abstract

Schizophrenia is associated with heterogeneous course of positive and negative symptoms. In addition, reduced motor activity as measured by wrist actigraphy has been reported. However, longitudinal studies of spontaneous motor activity are missing. We aimed to explore whether activity levels were stable within and between psychotic episodes. Furthermore, we investigated the association with the course of negative symptoms. In 45 medicated patients, we investigated motor behavior within a psychotic episode. In addition, we followed 18 medicated patients across 2 episodes. Wrist actigraphy and psychopathological ratings were applied. Within an episode symptoms changed but activity levels did not vary systematically. Activity at baseline predicted the course of negative symptoms. Between two episodes activity recordings were much more stable. Again, activity at the index episode predicted the outcome of negative symptoms. In sum, spontaneous motor activity shares trait and state characteristics, the latter are associated with negative symptom course. Actigraphy may therefore become an important ambulatory instrument to monitor negative symptoms and treatment outcome in schizophrenia.

## Introduction

Schizophrenia is characterized by positive symptoms, negative symptoms, and disorganization. Furthermore, cognitive and motor symptoms have been identified as relevant symptom clusters of schizophrenia. In fact, the current DSM5 concept of psychoses proposed eight dimensions of psychopathology to be assessed in subjects with schizophrenia, including motor symptoms (1). Schizophrenia is further associated with particular heterogeneity in course and outcome (2, 3). Generally, symptoms ameliorate during the course of a psychotic episode; however, negative symptoms do not respond as well to treatment as positive symptoms and disorganization too (4, 5). In longitudinal studies over years, negative symptoms generally tend to be stable (5, 6). However, findings of a recent meta-analysis suggest that negative symptoms may improve to a greater extent than what has previously been assumed (7).

Motor signs in schizophrenia include catatonia, neurological soft signs, psychomotor slowing, and extrapyramidal symptoms, i.e., abnormal involuntary movements, akathisia, and parkinsonism (8, 9). These motor symptoms are prevalent throughout the course of the disorder and may be affected by antipsychotic treatment. Particularly, gross motor behavior is important in schizophrenia; it is linked with medication, symptom dimensions, and relevant for physical health (10, 11). Spontaneous motor activity may be objectively assessed with continuous wrist actigraphy (12). A number of parameters can be calculated from actigraphy data, such as the activity level (AL; activity counts per hour), the movement index (MI; percentage of active periods), or the average duration of immobility periods (12). However, it was the AL that was associated with negative syndrome severity (13–15) and neuroimaging markers (16–18). In general, schizophrenia patients have reduced levels of daytime activity compared to healthy control subjects (15, 17, 19, 20). One study performed actigraphic recordings for seven consecutive days (19). Their graphs indicate rather stable measurements throughout a week, even though no statistical test had been applied to explore temporal fluctuations. Actigraphy has been used for cross-sectional comparisons of spontaneous motor behavior in schizophrenia; however, no study has addressed the longitudinal course within or even between psychotic episodes. Studies on motor abnormalities in first episode and chronic schizophrenia indicate that most motor symptoms including hypokinesia are attenuated by antipsychotic treatment within 4 weeks (21, 22). Therefore, we could assume that spontaneous motor activity is subject to changes within an episode. The longitudinal course of motor signs over more than a few weeks has only rarely been addressed. Few longitudinal studies included finger tapping and found no changes over time (23). Docx et al. report amelioration of catatonia and neurological soft signs in stable patients over 1 year (24). Likewise, neurological soft signs tend to decrease within the first year in schizophrenia (25).

The current study aimed at testing whether spontaneous motor activity would change during the course of a psychotic episode as well as during the course between two psychotic episodes. Furthermore, we wanted to investigate whether spontaneous motor activity at baseline would predict the course of symptoms in schizophrenia. The literature suggests wide distribution of activity levels between subjects with schizophrenia (15, 17). We hypothesized that a considerable proportion of the variance was stable over time (trait motor activity) and that some of the variable motor activity was state dependent and linked to negative symptoms of schizophrenia.

## Materials and Methods

### Participants

Participants were recruited from the inpatient and outpatient department of the University Hospital of Psychiatry, Bern, Switzerland. This investigation includes data from three different studies that all used the same actigraphy procedures. Diagnoses were given after thorough clinical examination and review of all case files by board certified psychiatrists. The local mental health care system ensures that most of the patients are admitted to our clinic in every psychotic episode requiring inpatient treatment, allowing the collection of reliable diagnostic information. In a proportion of studies that contributed data to this analysis, the diagnoses were ascertained by structured clinical interviews (both SCID and MINI; 27% of cases). Patients were excluded in case of substance abuse other than nicotine, medical conditions affecting physical activity (e.g., injuries such as fractures or ligament ruptures and conditions such as idiopathic parkinsonism, arthrosis, or rheumatism) and epilepsy. Physical activity was recorded approximately 2 weeks after admission to the psychiatric department (24 h at each assessment time point). At the same time, psychopathology was assessed using the positive and negative syndrome scale (PANSS) (20). In the within-episode sample, actigraphy and PANSS assessments were repeated after the acute symptoms had been alleviated. In the between episodes sample, assessments were repeated within the first 2 weeks of the subsequent inpatient treatment due to a psychotic episode. Episode and inter-episode interval have been defined according the practice guidelines developed by the American Psychiatric Association codified a three-phase model of schizophrenia disease course, with the recognition that these phases “merge into one another without absolute, clear boundaries between them” (26). According to this model, the “acute phase,” characterized by florid psychosis and severe positive and/or negative symptoms, which is followed by a “stabilization phase,” during which symptoms recede and decrease in severity, and a subsequent “stable phase” with reduced symptom severity and relative symptom stability. In detail, the stable phase reflects the inter-episode interval while the acute phase including the stabilization phase has been defined as episode and within-episode interval (26, 27). Chlorpromazine equivalents (CPZ) of the current antipsychotic pharmacotherapy were calculated according to Woods (21). The protocols of the studies applying wrist actigraphy in schizophrenia spectrum disorders had been approved by the local ethics committee and participants provided written informed consent prior to study inclusion.

#### Within-episodes sample

In total, 45 patients (28 men and 15 women) were included with an average period between measurements of 42 days. Mean age was 36.9 ± 9.5 years, mean duration of illness 11.1 ± 10.7 years. Patients experienced 6.8 ± 6.6 episodes. The majority was treated with antipsychotics (94%; aripiprazole, clozapine, quetiapine, risperidone, olanzapine, haloperidol, amisulpride, zuclopenthixol, and flupenthixol), most of them atypical (91%).

#### Between episodes sample

In total, 18 patients (13 men and 5 women) were included with an average period between episodes of 639 days (4 subjects of the within-episode sample were also included in the between episode sample). Mean age was 34.7 ± 10.5 years, mean duration of illness 8.7 ± 8.0 years. Patients experienced 6.9 ± 7.2 episodes. All patients received antipsychotic treatment (aripiprazole, clozapine, quetiapine, risperidone, olanzapine, haloperidol, and amisulpride) with predominantly atypical antipsychotics (index episode 83%, later episode 94%).

### Actigraphy

Participants wore an actigraph (Actiwatch, Cambridge Neurotechnology, Inc., Cambridge, UK) for 24 consecutive hours at the wrist of the non-dominant arm. The piezoelectronic sensor converts acceleration into movement counts. Data were sampled in 2 s intervals. Participants provided sleep log information and information on recording pauses (due to showering or bathing). Only the data collected during wakeful periods of the 24 h recording time were analyzed. Activity counts were averaged to provide the AL in counts/h.

### Statistical analyses

Positive and negative syndrome scale scores were used to calculate two factors of negative symptoms as in previous studies (15, 28, 29): the expressivity factor including items N1 (blunted affect) and N6 (lack of spontaneity and flow of conversation) and the avolition factor including items N2 (emotional withdrawal) and N4 (passive/apathetic social withdrawal). Longitudinal comparisons of clinical and actigraphic parameters were calculated using paired *T*-tests. To test the explained variance, we entered AL baseline as predictor of a linear regression model of AL post. In both cohorts, we detected wide variability of AL changes. Using Pearson correlations, we explored associations between AL at baseline and clinical parameters at baseline and in the longitudinal course. Next, we aimed at testing groups according to their baseline AL values. In order to test the longitudinal course of PANSS scores according to baseline motor activity, we defined groups applying the 33rd and 66th percentile of the baseline AL in both cohorts. Group comparisons (low, medium, and high AL) were conducted using repeated measures ANOVAs. *Post hoc* analyses were Sidak corrected for multiple comparisons. All analyses were conducted with SPSS22.

## Results

### Within episodes

Within psychotic episodes longitudinal AL changes were wide-ranged. Paired *T*-tests of AL baseline and AL post indicated no change (see Table 1). However, only 28% of the patients had stable AL, i.e., AL changed by <20%. AL post ranged 35–272% of the AL at baseline. In contrast, PANSS positive and PANSS total scores were attenuated, a trend for improvement was detected in PANSS negative scores (see Table 1). Interestingly, baseline AL inversely correlated at trend level with% of change of activity (*r* = −0.286; *p* = 0.063), indicating that the less patients move at baseline the more likely AL changes within episodes. However, the magnitude of change from baseline AL was not predictive of the longitudinal course of PANSS scores (*r*-range: −0.17–0.10).

**Table 1:** **Paired *T*-tests within episode**.

	Baseline	Post	*T*	df	*p*	*r*
AL (counts/h)	13735 ± 7274	13544 ± 8128	0.2	42	0.870	0.52
PANSS positive	17.1 ± 5.8	13.1 ± 4.8	4.3	42	<0.001	0.34
PANSS negative	18.4 ± 6.8	16.4 ± 6.2	1.7	42	0.099	0.30
PANSS avolition	5.3 ± 2.2	4.9 ± 2.4	0.8	42	0.406	0.10
PANSS expressivity	5.5 ± 3.0	5.0 ± 2.3	1.4	42	0.181	0.56
PANSS total	69.5 ± 16.6	59.0 ± 15.9	3.4	42	0.001	0.24
CPZ (mg)	543 ± 401	531 ± 435	0.2	42	0.807	0.69

**Figure 1:**
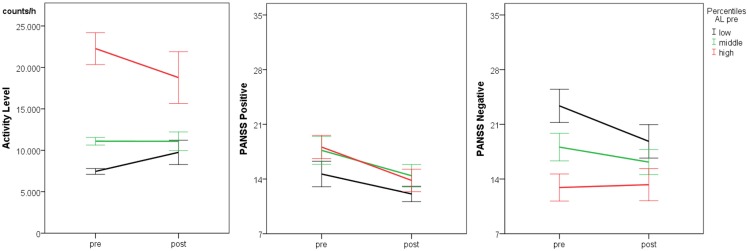
**Within-episode course of activity and psychopathology (*n* = 45) lines indicate mean ± SE**.

Activity level at baseline predicted AL post (*R*^2^ = 0.26, *F* = 15.0, *p* < 0.001). AL baseline correlated with PANSS negative baseline (*r* = −0.53, *p* < 0.001). Furthermore, AL post correlated with PANSS negative at both time points (PANSS negative baseline: *r* = −0.39, *p* = 0.011; PANSS negative post: *r* = −0.35, *p* = 0.021). Therefore, we explored whether AL baseline would indicate longitudinal changes in psychopathology. AL baseline was used to define three groups based on percentile rankings (33rd and 66th percentiles), i.e., low, medium, and high AL baseline. The three patient groups did not differ in terms of age (*F* = 1.3, df = 2, *p* = 0.294), duration of illness (*F* = 1.4, df = 2, *p* = 0.258), number of episodes (*F* = 1.2, df = 2, *p* = 0.311), CPZ (*F* = 0.01, df = 2, *p* = 0.992), and the time between measurements (*F* = 0.5, df = 2, *p* = 0.609). Time effects were strong for PANSS positive and total scores, indicating attenuation of symptoms during the episode (see Table 2; Figure 1). Significant group × time interactions were detected only for the measures of the negative syndrome: PANSS negative scores, avolition, and expressivity scores decreased significantly only in the group with low AL at baseline (*T* = 3.4, *p* = 0.005; *T* = 2.7, *p* = 0.019; *T* = 3.2, *p* = 0.006), while in the other groups negative syndrome scores remained stable.

**Table 2:** **Repeated measures ANOVAs within episode**.

		AL baseline group			Time		Group		Time × group	
		Low	Medium	High	*F*	*p*	*F*	*p*	*F*	*p*
AL	Baseline	7742 ± 1178	11077 ± 1469	22577 ± 6016	0.3	0.863	47.2	<0.001	0.8	0.455
	Post	9096 ± 4684	11329 ± 4174	20366 ± 9781	
PANSS positive	Baseline	14.5 ± 4.9	19.5 ± 6.1	17.3 ± 5.7	17.7	<0.001	5.1	0.011	0.03	0.967
	Post	10.8 ± 3.6	15.3 ± 5.0	13.0 ± 4.8	
PANSS negative	Baseline	21.9 ± 6.9	19.3 ± 5.6	13.9 ± 5.7	3.3	0.078	4.1	0.025	3.5	0.039
	Post	15.8 ± 5.7	18.5 ± 7.4	14.8 ± 5.2	
PANSS avolition	Baseline	6.2 ± 2.6	5.6 ± 1.9	4.1 ± 1.6	0.9	0.357	1.5	0.230	4.5	0.017
	Post	4.0 ± 2.3	5.7 ± 2.4	5.0 ± 2.3	
PANSS expressivity	Baseline	7.5 ± 3.0	5.3 ± 2.8	3.7 ± 2.0	2.3	0.134	5.2	0.010	4.8	0.014
	Post	5.4 ± 2.2	5.5 ± 2.7	3.9 ± 1.9	
PANSS total	Baseline	69.8 ± 12.9	76.9 ± 20.5	61.4 ± 11.7	11.9	0.001	4.6	0.016	1.1	0.339
	Post	53.6 ± 15.4	66.2 ± 16.0	56.6 ± 14.3	

*AL: high > low (*p* < 0.001) and high > medium (*p* < 0.001) *post hoc* Sidak tests*.

### Between episodes

**Figure 2:**
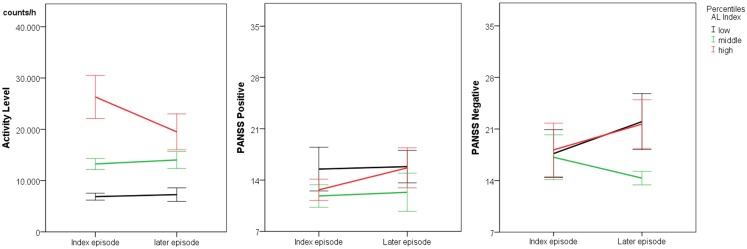
**Between episode course of activity and psychopathology (*n* = 18) lines indicate mean ± SE**.

Between episodes, AL was more stable than within episodes. In fact, 50% of the patients had AL changes of ±20%. AL at the later episode ranged 40–167% of AL at the index episode. Likewise, PANSS scores were similar between episodes (see Table 3), except a trend for deterioration in the PANSS avolition score. AL at index episode predicted AL at the later episode (*R*^2^ = 0.65, *F* = 30.0, *p* < 0.001). Neither AL at index episode nor AL at a later episode correlated significantly with PANSS scores. Still, when the group was divided according to AL at index episode (low, medium, high AL), we detected a significant group effect on PANSS negative scores, as well as a trend to a time × group interaction (see Table 4; Figure 2). Negative syndrome scores increased between episodes in patients with high AL at index episode (*T* = −2.6, *p* = 0.047) and at trend level also in patients with low AL at index episode (*T* = −2.1, *p* = 0.095). Likewise, avolition scores increased at trend level in the group with high AL at index episode (*T* = −2.7, *p* = 0.052). In the group with low AL at index episode, we found single PANSS negative items to increase over time at trend level: N1 (blunted affect, *p* = 0.076) and N3 (poor rapport, *p* = 0.076). In the group with high AL at index episode, we found trends for changes of the items N2 (emotional withdrawal, *p* = 0.070) and N6 (lack of spontaneity, *p* = 0.070).

**Table 3:** **Paired *T*-tests between episodes**.

	Index episode	Later episode	*T*	df	*p*	*r*
AL (counts/h)	15472 ± 10152	13591 ± 7474	1.3	17	0.203	0.81
PANSS positive	13.3 ± 5.1	14.6 ± 6.0	−1.4	17	0.183	0.77
PANSS negative	17.7 ± 7.6	19.6 ± 7.7	−1.1	17	0.274	0.67
PANSS avolition	4.1 ± 2.0	5.5 ± 3.2	−2.1	17	0.051	0.53
PANSS expressivity	5.5 ± 3.1	6.1 ± 2.9	−1.0	17	0.351	0.69
PANSS total	60.0 ± 16.1	65.4 ± 16.1	−1.2	17	0.253	0.29
CPZ (mg)	440 ± 305	608 ± 387	−1.8	17	0.092	0.36

**Table 4:** **Repeated measures ANOVA between episodes (*n* = 18)**.

		AL index group			Time		Group		Time × group	
		Low	Medium	High	*F*	*p*	*F*	*p*	*F*	*p*
AL	Index	6870 ± 1669	13241 ± 2623	26305 ± 10256	2.4	0.143	13.0	0.001	4.1	0.038
	Later	7263 ± 3211	14016 ± 4016	19495 ± 8567	
PANSS positive	Index	15.5 ± 7.3	11.8 ± 3.8	12.7 ± 3.6	1.9	0.189	0.7	0.522	0.86	0.442
	Later	15.8 ± 5.4	12.3 ± 6.4	15.7 ± 6.7	
PANSS negative	Index	17.7 ± 7.9	17.2 ± 7.4	18.2 ± 8.9	1.6	0.231	4.1	0.025	2.9	0.088
	Later	22.0 ± 9.3	14.3 ± 2.3	21.7 ± 8.1	
PANSS avolition	Index	4.7 ± 2.7	4.0 ± 2.0	3.4 ± 0.9	4.8	0.047	0.6	0.539	1.7	0.217
	Later	6.3 ± 4.1	3.8 ± 2.0	6.2 ± 2.7	
PANSS expressivity	Index	5.5 ± 2.9	5.8 ± 3.0	5.2 ± 3.8	0.8	0.396	0.1	0.880	1.4	0.273
	Later	7.0 ± 3.6	5.0 ± 1.2	6.0 ± 3.4	
PANSS total	Index	62.3 ± 17.9	55.3 ± 17.5	62.3 ± 15.3	1.3	0.275	1.5	0.264	0.3	0.751
	Later	69.5 ± 15.4	55.7 ± 13.7	71.0 ± 16.9	

*AL: high > low (*p* < 0.001) and high > medium (*p* = 0.028) *post hoc* Sidak tests*.

## Discussion

The present study explored the longitudinal course of spontaneous motor activity in schizophrenia. Results indicate considerable variance within psychotic episodes and stability of activity levels between episodes. Furthermore, within-episodes baseline activity predicted the outcome of negative symptom severity. Therefore, our hypotheses were confirmed: spontaneous motor activity in schizophrenia has trait and state characteristics; the latter are associated with the negative syndrome.

### Within-episode variance

Activity levels for the whole group demonstrated considerable variance, only 26% of the variance of AL post were predicted by AL at baseline. When we split the sample into three similar sized groups according to the baseline AL, we found no changes over time; instead, group differences remained throughout the episode. This stability of motor activity is congruent with the observations over 1 week (19). However, other motor signs in schizophrenia are attenuated by antipsychotic treatment. This is true for abnormal involuntary movements and neurological soft signs but also for hypokinesia, parkinsonism, and catatonia (21, 22, 25). Particularly, when hypokinetic movement disorders were ameliorated, we could expect an increase in AL within an episode. Our findings fail to support this notion. As noted above, there was considerable variance in the longitudinal course of AL within an episode. Peralta and Cuesta reported in their sample of 100 unmedicated first episode patients that 21% had parkinsonism and 18% catatonia at baseline (30). The two hypokinetic movement disorders demonstrated distinct courses with antipsychotic treatment; parkinsonism increased and catatonia declined within 4 weeks. Interestingly, for both syndromes, cases were identified who had drug emergent, drug responsive, and drug irresponsive courses (30). Therefore, patients can present with very different courses of movement disorders, which may explain our finding of stable AL in the paired *T*-test and only 26% of explained variance between assessments.

Activity level at baseline separated the groups according to negative syndrome severity. As in previous studies, lower AL was associated with increased negative syndrome scores (13–15). Furthermore, the groups differed in the course of negative syndrome. Patients in the low AL group experienced a pronounced decrease of negative syndrome scores within the episode, while the other groups did not. This finding was true for both, the avolition and the expressivity component of the PANSS negative syndrome score. Baseline AL may therefore predict who will have a higher probability of reduced negative symptoms with treatment. In line with the literature, within episodes in general PANSS positive and total scores decreased more than negative scores with antipsychotic treatment (4, 5, 31). In contrast, the avolition score remained stable (5, 32). Therefore, avolition may represent the invariant component of negative symptoms while other negative symptoms tend to improve (32). Interestingly, specifically deficits of “action-orientation” (slowing of the initiation of fine motor movements) have been associated with avolition (29). Likewise, problems of initiation of goal directed behavior might have led to psychomotor slowing in our study. Avolition is a critical component of the negative syndrome in schizophrenia, linked to impaired motivation and poor functional outcome (33). Measures of avolition are correlated with reduced AL in schizophrenia (15, 34). Because actigraphy is a well-accepted, ambulatory, and non-invasive way of obtaining objective data on spontaneous movement, it should be applied in larger pharmaceutical trials on negative symptoms in schizophrenia. At the very least, it could help to identify subjects at baseline with increased likelihood of responding to treatments for negative symptoms.

### Between episodes stability

Between two psychotic episodes AL was much more stable (65% explained variance) than within an episode. The time × group interaction indicated regression to the mean for AL in the longitudinal course. Our results argue for a trait component in AL in schizophrenia. Data on the long-term course of other motor phenomena are scarce and produced mixed results. Studies on neurological soft signs found subjects with increasing and subjects with decreasing symptoms (24, 25). The patients with increasing soft signs had poorer overall outcome (25). Catatonia was found to be reduced within 1 year even in chronic schizophrenia, but parkinsonism and motor retardation were unchanged (24). In contrast, cross-sectional reports of motor function in schizophrenia (chronic vs. first episode) noted generalized deterioration with ongoing course (35). Still, longitudinal studies of motor behavior during multiple episodes are missing.

Long-term studies starting in the first psychotic episodes indicate that considerable proportions of negative symptoms remain unchanged over several years (5, 6, 32). Again, avolition was most stable among the negative symptoms (5, 32). In our study, both positive and negative symptom scores displayed no time effect in the whole group, which may be accounted for by the small sample size. Still, correlations between index and later episode were strong for both syndromes. However, the avolition score indicated overall deterioration of this component of negative symptoms. In patients with high AL at baseline, single PANSS negative items emotional withdrawal (N2) and lack of spontaneity (N6) indicated deterioration at trend level in this group. Again, we noted an effect of AL on negative syndrome scores with a significant group effect and a group × time interaction at trend level. Here, patients with higher AL at index episode increased their negative syndrome scores between episodes. This is particularly interesting as the negative syndrome scores at baseline were similar between groups. Thus, only our measure of spontaneous motor activity had predictive value for negative syndrome scores. Again, results must be interpreted with caution due to the small groups.

### Limitations

This was a truly naturalistic study design, which is not able to detect longitudinal changes associated with specific drug effects. There are various effects that may have contributed to the observed changes. Medication may have heterogeneous effects on motor activity (36). The observation period could not be standardized. In addition, the sample sizes were small, particularly for the inter-episode comparison. Thus, we may have missed effects due to type-II errors. In addition, diagnoses were given after thorough clinical psychiatric examination and chart review; however, structured clinical interviews (both SCID and MINI) were only applied in a proportion of (27% of cases) patients. We assessed negative symptoms with the PANSS negative syndrome scale and PANSS factors for avolition and expressivity. Additional negative scales such as the scale for the assessment of negative symptoms (SANS) (37) or the clinical assessment interview for negative symptoms (CAINS) (38) were not applied. Finally, future studies could include measures of motor syndromes such as catatonia, neurological soft signs, and parkinsonism to account for differential effects of medication on motor signs (30). In sum, we think our results are encouraging for large randomized controlled trials focusing on negative symptoms in schizophrenia.

## Conclusion

Spontaneous gross motor behavior in schizophrenia shares trait and state characteristics. Within-episodes motor activity varies with negative syndrome scores, while between psychotic episodes motor activity remains stable. Wrist actigraphy should be considered as objective ambulatory non-invasive instrument to monitor the effects of treatment on negative symptoms.

## Author Contributions

SW and HH designed the study. SW wrote the protocol. LR, NR, KS, and SW recruited participants and performed assessments. SW performed the data analyses. All authors interpreted and discussed the findings. SW wrote the first draft of the manuscript. All authors contributed to the final version of the manuscript.

## Conflict of Interest Statement

The authors declare that the research was conducted in the absence of any commercial or financial relationships that could be construed as a potential conflict of interest.
